# Expression of MMP-9 decreases metastatic potential of Chondrosarcoma: an immunohistochemical study

**DOI:** 10.1186/s12891-017-1920-7

**Published:** 2018-01-09

**Authors:** Dominik Malcherczyk, Thomas J. Heyse, Bilal F. El-Zayat, Vanessa Kunzke, Roland Moll, Susanne Fuchs-Winkelmann, Jürgen R. J. Paletta

**Affiliations:** 1 Center for Orthopedics and Trauma Surgery, University Hospital Marburg, Baldingerstrasse, 35043 Marburg, Germany; 2 Department of Pathology, University Hospital Marburg, Marburg, Germany

**Keywords:** Matrix metalloproteinases, MMP, Chondrosarcoma, Metastasis, Prognosis, Grading

## Abstract

**Background:**

Chondrosarcoma is the second most common primary malignant bone tumor. Because of their heterogeneity, with differences in invasive and metastatic behavior, it is important to identify biological markers that will allow for a more accurate estimation of prognosis in patients with these tumors.

Matrix metalloproteinases (MMP) play a crucial role in tumor progression, invasion and metastasis. The mechanism of tumor progression dependent of MMPs is complex and influences malignant transformation, angiogenesis and tumor growth at the primary and metastatic sites. The purpose of this study was to investigate immunohistochemicaly the influence of MMP-1, MMP-3, MMP-9 and MMP-13 expression on prognostic parameter in chondrosarcoma.

**Methods:**

We investigated tissue samples of 28 patients with chondrosarcoma. Immunohistochemical staining to evaluate the expression of MMP-1, MMP-3, MMP-9 and MMP-13 was performed. Subsequently, the expression level was correlated with metastatic potential, histological grading and overall survival in patients with this neoplasm.

**Results:**

In consideration of semi quantitative scoring 64% of chondrosarcoma were scored as positive for MMP-1, 46% for MMP-3, 61% for MMP-9. The specimens had shown no expression of MMP-13. High expression of MMP-9 was associated with better histological differentiation, decreased metastatic potential and favourable overall survival. No correlation was found for expression of MMP-1, MMP-3 or MMP-13.

**Conclusions:**

MMP-1, MMP-3 and MMP-9 are expressed in chondrosarcoma. Our findings suggest that the expression of MMP-9 is associated with clinical outcome parameters in chondrosarcoma.

## Background

Chondrosarcomas are malignant bone tumors that are characterized by the formation of cartilaginous neoplastic tissue. They are the second most common, accounting for 26% of all osseous malignances [1]. Chondrosarcomas are very heterogeneous group of tumors and range from locally aggressive tumors with almost no metastatic potential to high-grade malignances with a marked propensity to metastasize [2–4]. To reflect its biological behavior, chondrosarcoma has been differentiated based on the cellular and structural atypia into three grades [3]. Although the histological grading is frequently inconsistent compared to metastatic and invasive attitude in this chondroide malignant neoplasm therefore some other prognostic markers were examined [5–8]. However, none of them could win clinical recognition, so to estimate the aggressiveness and choose appropriate therapy for patients with chondrosarcomas more reliable tumor markers are needed.

The degeneration and penetration of extracellular matrix (ECM) and basal lamina are important steps in invasive growth and metastasis development in tumors [9]. Matrix metalloproteinases (MMPs) are endopeptidases that play a crucial role in ECM degradation [10–12]. The family of MMPs consists of 23 members which are generally classified according to substrate specificity. Key importance in the degeneration of collagen, basement membrane and other proteins of extracellular matrix have especially MMP-1 and MMP-9. MMP-1 cleaves the majority subtypes of collagen. In this way accrued gelatine can be cleaved by MMP-9. As demonstrated in the study of Dreier et al., MMP-3 and MMP-13 have a key regulatory function, especially in the activation of MMP-9. [10–14].

A number of studies have demonstrated correlation between the expression of different MMPs and the potential of invasive growth, poor tumor differentiation, stage of cancer, prognosis or development of metastases in many human cancers [15–18]. In most of these cancers MMPs expression correlated with their aggressiveness.

Despite the tumorigenic function of most of the members of the MMP-family, recent studies demonstrated, that some MMPs negatively influence tumor cell progression and their ability to metastasize [19, 20]. Moreover, several MMPs might have dual roles either promoting or suppressing tumor genesis depending on the type of cell in with they are expressed [21]. Also in human chondrosarcoma presence of MMPs was demonstrated, but to the best of our knowledge there is no study that investigated the influence of the expression of MMPs on the metastatic potential of these tumors.

The purpose of this study was to investigate the immunohistological expression of MMP-1, −3, −9, −13 in human chondrosarcoma and its correlation with metastatic potential as well as histological grading and survival of the patients. It was hypothesized, that MMP expression would positively correlate with higher tumor grading, metastasis and a shorter survival.

## Methods

### Tumor samples

Procedures followed were in accordance with the ethical standards and with the Helsinki Declaration of 1964, and its later amendments and comparable ethical standards. The study was approved by local ethics committee.

Medical records of 36 patients with chondrosarcoma who had been diagnosed and treated in the authors’ center were retrospectively explored. Twenty-eight patients whose tissue specimens were available were included in this study. Thirteen patients were male and 15 patients were female. Median age was 47.5 years (range, 13–82). The median follow-up was 70 months. Twenty-one specimens were classified as conventional chondrosarcoma, two as dedifferentiated chondrosarcoma, two as extraskeletal chondrosarcoma, and one as periostal, mesenchymal and clear-cell chondrosarcoma each. According to the size and shape of the cell nucleus, count of mitosis and number of cells the tumors were divided into three differentiation grades. Eleven cases were categorized as grade I (39.3%), 12 as grade II (42.9%) and five as grade III (17.9%) chondrosarcoma. Twenty-four patients were treated with surgical resection, in some cases combined with chemotherapy. Two patients underwent radiotherapy (one combined with chemotherapy). Because of general condition two patients received no therapeutic treatment at all. Twelve patients (42%) developed metastases, including four patients with known metastases at the time of diagnosis.

### Immunohistochemical staining and staining analysis

The selected, paraffin-embedded tissues were immunostained with mouse monoclonal antibodies against MMP-1 (Clone: 2Q519, dilution 1:750), US Biological, rabbit monoclonal antibodies against MMP-3 (Clone: EP1189Y, dilution 1:250), MMP-9 (Clone: EP1254, dilution 1:250) and MMP-13 (Clone: EP1263Y, dilution 1:250), Epitomics using the Streptavidin-method. The sections were dewaxed by xylene rinsing and rehydrated gradually through graded alcohols. The slides were rinsed with distilled water and placed in a jar with a 1:10 diluted citrate buffer at a pH of 6.0. The jar was heated in a steamer for 10 min. After cooling and rinsing with water, TBS-HCl-TWEEN and blocking the peroxidase, the tissues were incubated with mouse (MMP-1, MMP-13) or rabbit (MMP-3, MMP-9) normal serum for 30 min and with primary antibody against MMP-1, MMP-3, MMP-9 or MMP-13 overnight at 4 °C. After further incubation with biotinylated anti-rabbit or anti-Mouse antibody the specimens were treated with RT-streptavidin-complex for 30 min. Visualisation was performed with diamiobenzidine and hydrogen peroxide.

The stained slides were rinsed with distilled water and stained for 5 min with haemalaun as counterstain. Finally, the sections were rinsed with water and treated with graduated-density alcohol and with xylol. As positive controls skin tissues were used.

The immunohistochemical results were evaluated by an independent investigator who was unaware to the clinical information.

The amount of positive and negative cells was counted. Following, we determined the percentage of MMP-positive cells and we graduated these results semi quantitatively. Similar to other authors, [22, 23] MMP-3, MMP-9 and MMP-13 samples were scored negative when stained cells were less than 25% and positive when stained cells where more than 25%. For MMP-1 negative when stained cells were less than 75% and positive when there were more than 75% of stained cells. Different cut of value for MMP-1 was chosen because of different and mostly higher basic expression of this protein in many tumors.

### Statistical analysis

The data was analysed with SPSS analytic software. The ANOVA test was used to analyze the relationship between MMP expression and histological grade. Kaplan-Meier method and Long Rank Test were used to estimate the survival and metastatic potential. For all tests *p* < 0.05 was considered to be statistically significant.

## Results

**Fig. 1:**
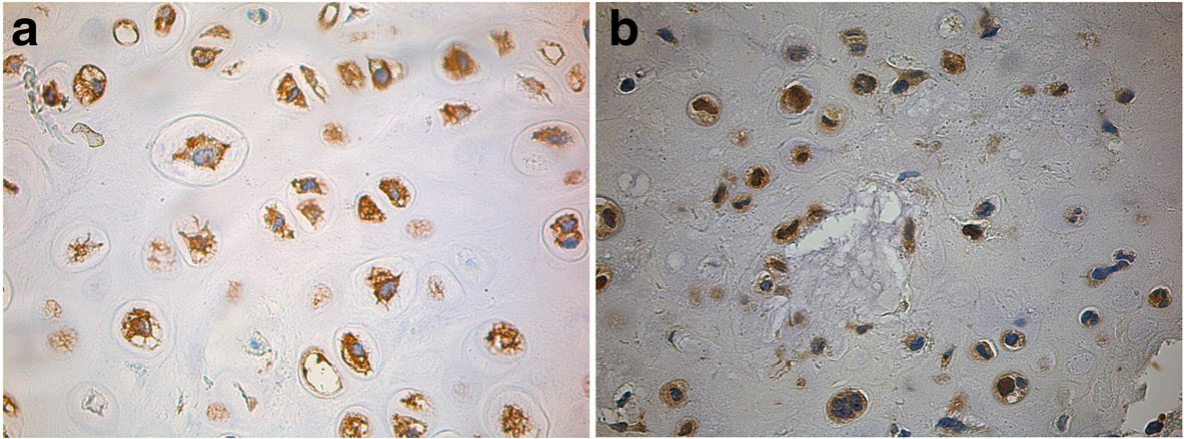
Examples of positive staining for MMP-1 (**a**) and MMP-9 (**b**) in two grade 2 chondrosarcomas

Of the 28 tumors included, most of the chondrosarcoma samples expressed the MMP-protein-variants explored in our study. In consideration of semiquantitative scoring 64% of chondrosarcoma were scored as positive for MMP-1, 46% for MMP-3, 61% for MMP-9 and no for MMP-13 (Fig. 1).

### Association of the expression of MMPs and metastatic potential and survival in chondrosarcoma

**Fig. 2:**
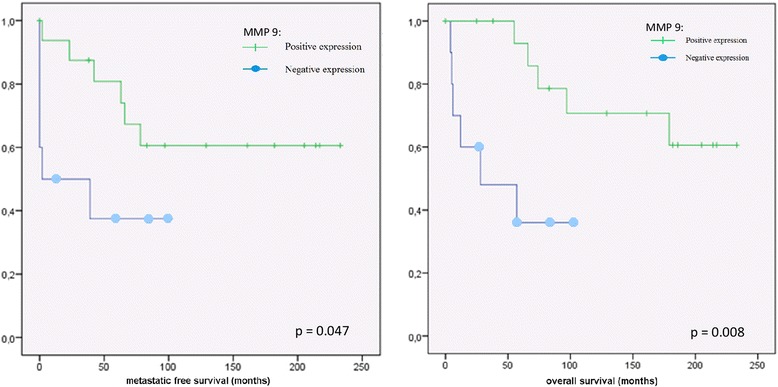
Kaplan-Meier analysis of the expression of MMP-9 (dash: positive expression; spot: negative expression) and metastatic free survival respectively of overall survival in chondrosarcoma

Positive expression of MMP-9 was associated with increased metastatic free survival time of patients with chondrosarcoma (*p* = 0.047) in Kaplan-Maier analysis (Fig. 2). After 5 years, 19% of chondrosarcoma with high MMP-9 expression developed metastasis, compared to 62% with lower MMP-9-expression.

**Fig. 3:**
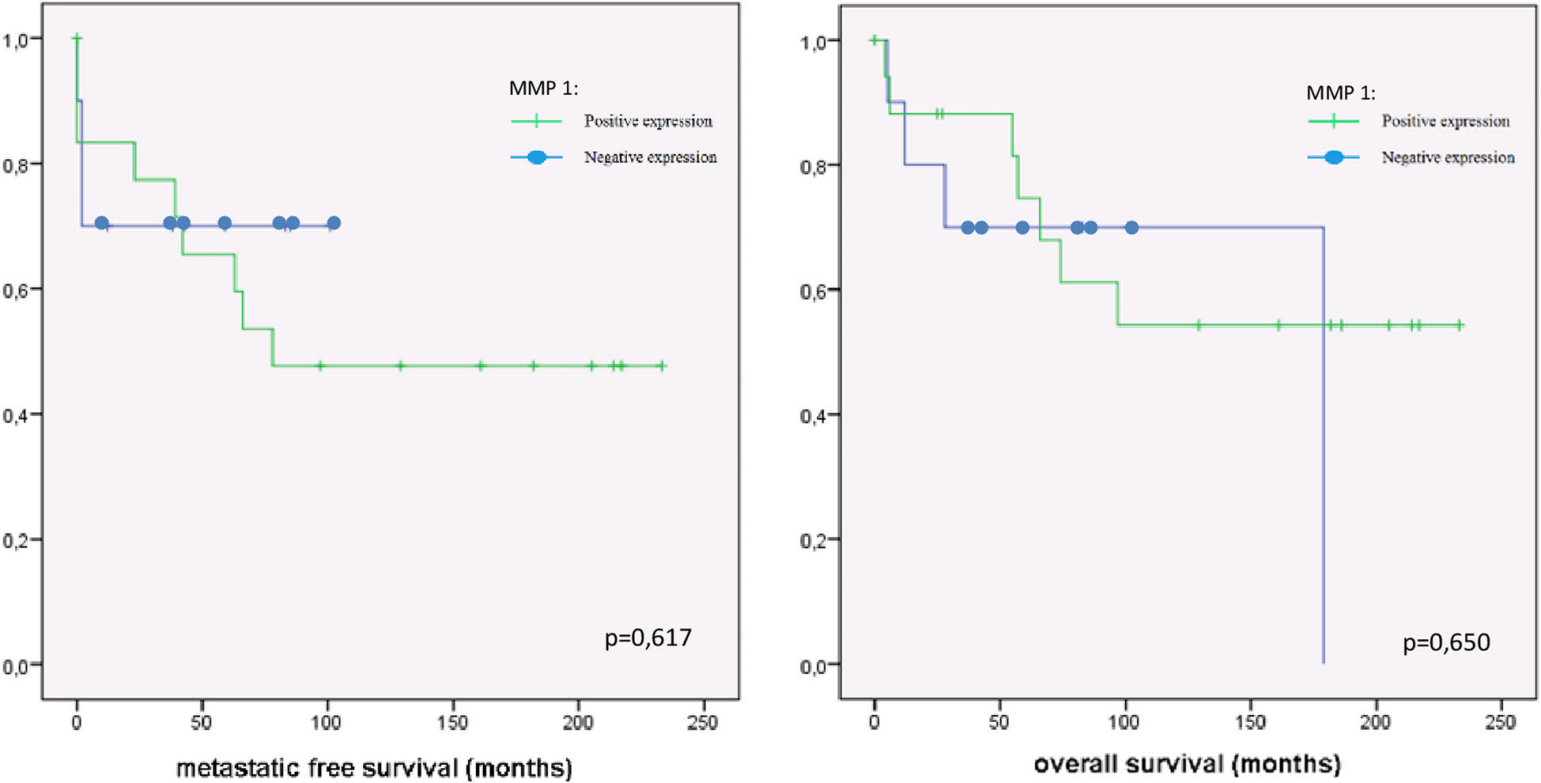
Kaplan-Meier analysis of the expression of MMP-1 (dash: positive expression; spot: negative expression) and metastatic free survival respectively of overall survival in chondrosarcoma

**Fig. 4:**
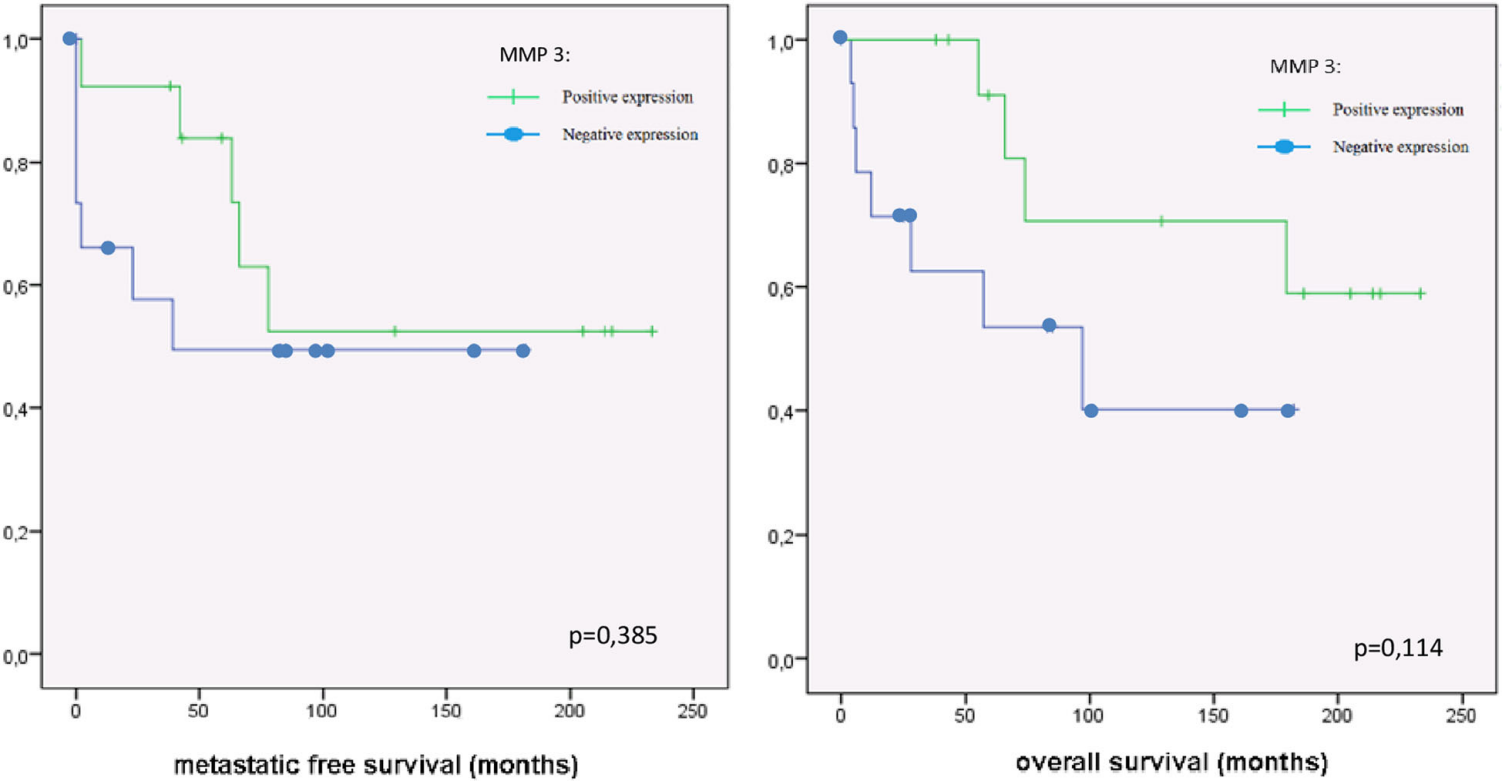
Kaplan-Meier analysis of the expression of MMP-3 (dash: positive expression; spot: negative expression) and metastatic free survival respectively of overall survival in chondrosarcoma

The positive correlation of metastatic potential and the expression of MMP-9 was reflected in the survival analysis in patients with chondrosarcoma. High expression of MMP-9 correlate with better survival (*p* = 0.008). The 5-year survival time for patients with chondrosarcoma with positive MMP-9 expression was 87%, compared to 36% in chondrosarcoma with negative MMP-9 staining (Fig. 2). There was no significant correlation between the expression of MMP-1, MMP-3 or MMP-13 and metastatic potential or survival in chondrosarcoma (Figs. 3 and 4).

### Association of the expression of MMPs and grading in chondrosarcoma

There was significant correlation between the expression of MMP-9 and histological grading of chondrosarcoma (*p* = 0.011). Better differentiated chondrosarcoma (grade I and grade II) expressed more MMP-9 then high-grad chondrosarcoma with poorer differentiation (grade III). For MMP-1, MMP-3 and MMP-13, no relationship with grading could be shown.

## Discussion

This study is an effort to correlate MMP expression with grading, metastatic potential and ultimately survival in chondrosarcoma. Due to the function of MMP in other sarcoma it was hypothesized, that MMP expression would positively correlate with higher tumor grading, metastasis and shorter survival.

Indeed in this study MMP-1, −3, −9 were found in the majority of the chondrosarcoma specimens. However there was a positive association between higher expression of MMP-9 and the metastatic-free-survival in Kaplan-Meier analysis, only. Increased expression of this protein was favourable for the overall survival of patients with chondrosarcoma and correlated with lower histological grading in these tumors. No correlation of the expression of MMP-1, MMP-3 or MMP-13 with metastasis-free-survival, overall survival or histological grading was found.

Although, the initial hypotheses were not confirmed, the results of our examination are in accord with studies that demonstrate protective effect of elevated expression of MMP-9 and other MMPs in multiple cancers. In epithelial-myoepithelial salivary human gland cancer, high staining intensity of both MMP-9 and MMP-13 was associated with better overall-survival [24]. Furthermore decreased expression of MMP-2 and MMP-9 was shown in ileal carcinoids with liver metastases compared to tumors with metastatic free status. Down regulation of MMP-2 in the primary tumor revealed unfavourable outcome of the disease [25]. Also in canine mammary tumors the percentage of MMP-positive cells was higher in adenomas then in carcinomas [26]. Taken together it might be concluded that positive staining of MMP-9 may have a beneficial role in different tumors.

Interestingly, the results are different from those of some other studies examining expression of MMP in sarcomas. For example, the expression of various MMPs, such as MMP-1 and MMP-2 was associated with poor prognosis in osteosarcoma and soft tissue sarcoma [22, 27, 28]. Also in chondrosarcoma elevated expression-levels of MMPs indicated rather an aggressive behaviour of these tumors. Soederstrom et al. could show that chondrosarcoma expressed increased levels of MMP-13 and MMP-14 mRNAs (messenger Ribonuclein acids) when compared with non-malignant control samples. Some individual chondrosarcomas also exhibited elevated levels of MMP-1, MMP-7 and MMP-9 mRNAs [29]. In another study, correlation of elevated MMP-2 expression and tumor grading of chondrosarcoma was proven [30]. Scully et al. reported that a high ratio of MMP-1 mRNA to its tissue inhibitor (TIMP-1 [Tissue Inhibitor of Metalloproteinases 1]) mRNA correlated with invasiveness, aggressiveness and disease-free-survival in human chondrosarcoma [31]. In the study from Sugita et al. there was an association between histological grade and the expression level of some MMPs (MMP-2, −3, −13) or their inhibitor proteins (ADAMTSs [A Disintegrin And Metalloproteinase with Thrombospondin Motifs] and TIMPs). In this patient population no significant correlation of expression of these proteins and prognosis could be found [23].

In many other malignant tumors such as gastric cancer, renal carcinoma, breast carcinoma, rectal carcinoma and small cell lung cancer high MMPs expressions increase cancer invasion and metastasis [16–18, 32, 33].

Explanation for the divergent results, with respect to the MMP expression provides some recent examinations. The prevailing view that MMP promote metastasis and tumor growth has led to the use of small molecule inhibitors of proteases in animal models for treatment of cancer. However, several of these molecules were ineffective in clinical trials and in some cases even an acceleration of tumor progress was suggested [11]. The oncogenic and pro-metastatic role of MMPs was initially subscribed to their ability to degrade the components of extra-cellular matrix, but now they are known to have functions that extend far outside matrix remodelling. These proteases can impact every stage of tumor progression through processing of several growth factors, regulation of the apoptosis or via influencing angiogenesis. In some cases, these functions may lead to anti-tumorigenic effects.

Especially MMP-9 is strongly associated with anti-angiogenic effects, derived from its capacity to generate angiogenesis inhibitors (endostatin and tumostatin) and might play a protective role in some tumors [11, 34]. For example MMP-9 can in vivo increase the levels of endostatin in nude mice and therefore decreased vessel density and reduced tumor growth [35]. Also in chondrosarcoma, as demonstrated by Furumatsu et al. endostatin leads to reduced tumor growth [36]. On the other hand MMP-9 knock-out mice express less tumostatin. By the absence of tumostatin increased tumor growth could be observed in lung cancer cells [37].

Interestingly, recent large-scale genomic studies have shown that MMP’s could be genetically or epigenetically altered in various human malignant tumors. These mutations may lead to loss-of-function and enhanced progression of the cancer. In fact, several MMPs, including MMP-9, might have dual roles either promoting or suppressing tumorgenesis depending on the type of cell in which they are expressed [21].

As demonstrated above, MMP-9 can inhibit tumor development, mostly through influencing the angiogenesis, for example in the metastatic foci, of different neoplasm. Therefore it is necessary to perform more studies in chondrosarcoma, most suitable in cell lines, to investigate the interaction between MMP-9 and different angiogenic factors (e.g. endostatin or tumostatin) and to determine the impact of these interactions on tumor growth and progression in this neoplasm.

In our examination we included all chondrosarcoma subtypes, thus the heterogeneity of the samples is a limitation of this study. However, caused by the rare chondrosarcoma occurrence, the number of examined patients is not different compared to other studies and all these tumors have in common that they are malignancies producing chondroid matrix, which justifies putting them together.

## Conclusions

In conclusion, MMP-1, MMP-3 and MMP-9 are often expressed in chondrosarcoma. This study demonstrates that MMP-9 expression in chondrosarcoma is associated with a lower occurrence of metastases, lower grading and a better survival of patients with chondrosarcoma. It may be useful as a prognostic marker in these tumors.

## Abbreviations

ADAMTS: A Disitegrin and Metalloproteinase with Thrombospondin Motifs
ECM: extracellular matrix
MMP: Matrixmetalloproteinase
mRNA: messenger Ribonuclein acid
TIMP: Tissue Ihnhibitor of Metalloproteinases
